# Trematocine-derived antimicrobial peptides from the Antarctic fish *Trematomus bernacchaii*: potent antibacterial agents against ESKAPE pathogens

**DOI:** 10.3389/fmicb.2024.1447301

**Published:** 2024-08-07

**Authors:** Damiano Squitieri, Federica Massaro, Monica Mollica Graziano, Stefano Borocci, Margherita Cacaci, Maura Di Vito, Fernando Porcelli, Roberto Rosato, Francesca Ceccacci, Maurizio Sanguinetti, Francesco Buonocore, Francesca Bugli

**Affiliations:** ^1^Department of Basic Biotechnological Sciences, Intensive and Perioperative Clinics, Catholic University of the Sacred Heart, Rome, Italy; ^2^Department for Innovation in Biological, Agro-Food and Forest Systems (DIBAF), University of Tuscia, Viterbo, Italy; ^3^Institute for Biological Systems of Italian National Research Council (ISB-CNR), Secondary Office of Rome-Reaction Mechanisms c/o Department of Chemistry, La Sapienza University of Rome, Rome, Italy; ^4^Department of Laboratory Sciences and Infectious Diseases, A. Gemelli University Hospital Foundation IRCCS, Rome, Italy

**Keywords:** antimicrobial peptides, antimicrobial resistance, ESKAPE pathogens, membranolytic agents, multi-drug resistant bacteria

## Abstract

**Introduction:**

This study investigated the interaction with membrane mimetic systems (LUVs), bacterial membranes, the CD spectra, and the bactericidal activity of two designed trematocine mutants, named Trem-HK and Trem-HSK. Mutants were constructed from the scaffold of Trematocine (Trem), a natural 22-amino acid AMP from the Antarctic fish *Trematomus bernacchii*, aiming to increase their positive charge.

**Methods:**

The selectivity of the designed AMPs towards bacterial membranes was improved compared to Trematocine, verified by their interaction with different LUVs and their membranolytic activity. Additionally, their α-helical conformation was not influenced by the amino acid substitutions. Our findings revealed a significant enhancement in antibacterial efficacy against ESKAPE (*Enterococcus faecium, Staphylococcus aureus, Klebsiella pneumoniae, Acinetobacter baumannii, Pseudomonas aeruginosa*, and *Enterobacteriaceae* family) pathogens for both Trem-HK and Trem-HSK.

**Results:**

Firstly, we showed that the selectivity of the two new designed AMPs towards bacterial membranes was greatly improved compared to Trematocine, verifying their interaction with different LUVs and their membranolytic activity. We determined that their α-helical conformation was not influenced by the amino acid substitutions. We characterized the tested bacterial collection for resistance traits to different classes of antibiotics. The minimum inhibitory and bactericidal concentration (MIC and MBC) values of the ESKAPE collection were reduced by up to 80% compared to Trematocine. The bactericidal concentrations of Trematocine mutants showed important membranolytic action, evident by scanning electron microscopy, on all tested species. We further evaluated the cytotoxicity and hemolytic activity of the mutants. At 2.5 μM concentration, both mutants demonstrated low cytotoxicity and hemolysis, indicating selectivity towards bacterial cells. However, these effects increased at higher concentrations.

**Discussion:**

Assessment of *in vivo* toxicity using the *Galleria mellonella* model revealed no adverse effects in larvae treated with both mutants, even at concentrations up to 20 times higher than the lowest MIC observed for *Acinetobacter baumannii*, suggesting a high potential safety profile for the mutants. This study highlights the significant improvement in antibacterial efficacy achieved by increasing the positive charge of Trem-HK and Trem-HSK. This improvement was reached at the cost of reduced biocompatibility. Further research is necessary to optimize the balance between efficacy and safety for these promising AMPs.

## 1 Introduction

One of the most severe threats to modern public health is related to the antimicrobial resistance (AMR) establishment, with particular regard on bacterial opportunistic pathogens (O’Neill, 2016; Mba et al., 2022). This phenomenon occurs when genetic and phenotypic changes in bacteria cause the antibiotics to become less effective when used to treat infections. Recently, it was estimated that in 2019, on the basis of predictive models, there were 4.95 million (3.62–6.57) deaths globally associated with bacterial AMR (Murray et al., 2022). In 2017 (and in 2024 update), the World Health Organization (WHO) published a list of pathogens for which urgent action was needed: it includes pathogens of the ESKAPE group (Browne et al., 2020; WHO, 2024). These bacteria have garnered the acronym “ESKAPE” due to their remarkable ability to escape the antimicrobial actions of conventional antibiotics, making them responsible for a significant portion of healthcare-associated infections worldwide (Ayobami et al., 2022). Among all the different classes of antibiotics currently in use, β-lactams stand out as some of the most frequently prescribed (Kapoor et al., 2017). Regrettably, many strains of bacteria have developed β-lactamases, enzymes that deactivate β-lactam antibiotics by breaking down the characteristic 4-atom β-lactam ring by hydrolysis. These versatile enzymes have a broad range of substrates and are known as Extended Spectrum β-lactamases (ESBLs). According to Ambler’s classification, which categorizes β-lactamases based on structural similarities, there are four classes: three of them feature a serine residue in their active site, while the fourth class consists of metallo-β-lactamases (MBLs) that incorporate a Zinc ion in their active site (referred to as Group B β-lactamases) (Pandey et al., 2023). The majority of these MBLs are encoded by genes such as *bla*VIM, *bla*IMP, and *bla*NDM-1. Horizontal gene transfer is a key mechanism by which ESKAPE pathogens acquire and spread antibiotic resistance genes. The rise of pathogens that are resistant to existing antibiotics, making infections more difficult to treat and increasing the risk of deadly outbreaks, coincides with a declining pipeline of new antibiotic development (Hussain et al., 2021). Therefore, the design and development of new antimicrobial drugs to fight resistant infections is increasingly urgent for public health. Antimicrobial peptides (AMP) are possible candidates to help in solving this problem (Magana et al., 2020). These peptides are widely distributed in all organisms, from plants to bacteria, invertebrates, and vertebrates, as part of the innate immune responses (Koo and Seo, 2019; Cardoso et al., 2021; Li et al., 2022). Classification is often based on their structure, such as α-helices, β-sheets, or extended and loop structures, with α-helix AMPs typically displaying an amphipathic nature (Buonocore et al., 2019). These peptides primarily target the bacteria plasmatic membrane, and their selectivity, with respect to mammalian cell membrane, depends on primary and secondary structure and membrane charge density, considering the negative charge commonly found in bacterial cell walls. Their ability to target a broad spectrum of pathogens (bacteria, viruses, fungi, and parasites), including ESKAPE group, united with their relatively low likelihood of inducing resistance, makes them attractive candidates for developing novel antimicrobial treatments (Luo et al., 2023). In this paper, we investigate the biological activity of two mutants of trematocine (Della Pelle et al., 2020), an AMP identified from the red-blooded Antarctic fish *Trematomus bernacchii*. Antarctic species are an excellent source for novel biomolecules due to the peculiar environment where they live and to the specific adaptations that they have evolved. Some specific site mutations were designed to improve the bactericidal activity of the natural peptide. Firstly, we determined the interactions between these new AMPs and phospholipid vesicles of different composition designed to mimic both bacterial and mammalian cell membranes. Subsequently, we analyzed their structural characteristics and assessed their bactericidal efficacy against a large spectrum of clinical isolates (*n* = 50) belonging to the ESKAPE group. The different bacterial strains were firstly genotypically and phenotypically characterized for resistance traits to different classes of antibiotic drugs commonly used in clinical practice. Successively, we evaluated the *in vitro* toxicity of these AMPs on both mammalian cell lines and human erythrocytes. Finally, we conducted *in vivo* toxicity experiments using *Galleria mellonella* larvae model to preliminary evaluate the potential pharmacological applications of these peptides. This multifaceted approach not only enhances our understanding on the peptides’ properties but also provides valuable information for their future possible applications as new antibacterial drugs.

## 2 Materials and methods

### 2.1 Chemicals and peptides

All chemicals and solvents were purchased from Sigma-Aldrich. The lipids 1-palmitoyl-2-oleoyl-*sn*-glycero-3-phosphocholine (POPC) and 1-palmitoyl-2-oleoyl-*sn*-glycero-3-phospho-(1′-rac-glycerol) (POPG) were purchased from Avanti Polar Lipids (Alabaster, AL, USA).

Peptides Trem (FFGHLLRGIVSVGKHIHGLITG), Trem-HSK (WFFGKLLRGIVKVGKKIKGLIT) and Trem-HK (WFFGKLLRGIVSVGKKIKGLIT) were purchased from CASLO ApS, c/o Scion Technical University of Denmark with a purity >98%. Peptide concentrations were determined by absorption spectroscopy at 280 nm before each analysis.

### 2.2 Lipid vesicles preparation

The large unilamellar vesicles (LUVs) were composed of 100% POPC and a 70/30% (w/w) combination of POPC/POPG at a final concentration of 10 mM. The LUVs were prepared as previously described (Della Pelle et al., 2020). Briefly, specific amounts of lipids were dissolved in chloroform/methanol 9:1 (v/v). The solvent was then removed by rotary evaporation, and the samples were placed overnight under high vacuum. The lipid film was subsequently hydrated by adding 1 mL of buffer solution (10 mM phosphate buffer pH 7.4 with 150 mM NaCl and 0.8 mM EDTA) to obtain 10 mM lipid dispersion and subjected to 5 freeze–thaw cycles. The lipid suspensions were finally extruded through a polycarbonate membrane with pore of 100 nm using an Avanti Polar mini extruder. The LUVs obtained were used within 48 h from their preparation.

### 2.3 Partition constant determination

Peptide partitioning between a polar and apolar environment was studied by fluorescence spectroscopy, monitoring the increase of fluorescence of tryptophan (contained in the sequence of the two mutant peptides) upon increasing concentration of LUVs in the range between 0 to 1.1 mM.

Precisely, 1.0 μM solution of the peptide was titrated with LUVs of different compositions (POPC 100% and (70/30%) (w/w) POPC/POPG), and the fluorescence emission spectra were recorded between 315 and 400 nm with λ_exc_ = 290 nm. The measurements were performed with a scan speed of 200 nm/min, bandwidth for excitation and emission of 5 and 10 nm, respectively, and with a cross-oriented configuration of the polarizers (*pol_em_* = 0° and *pol_exc_* = 90°) to reduce scattering from vesicles. The background buffer emission was subtracted from each spectrum. Mole fraction partition coefficients (*K*_x_) were calculated from the fraction of the partitioned peptide (*f*_p_) as previously described according to the Wimley equation (Wimley, 2010):


$$f_{p}=\frac{K_{x}\;\left[L\right]}{W+K_{x}\;\left[L\right]}$$

Finally, the free energy of partition was calculated using the relationship:


$$\Delta G^{{}^{\circ}}=-\text{RTln}\;K_{x}$$

A Perkin Elmer LS 55 fluorometer was used for steady-state fluorescence measurements. The experiments were carried out at 25°C.

### 2.4 Quenching experiments

For all peptides, the fluorescence quenching was evaluated both in buffer solution, and in the presence of liposomes, by measuring the change of tryptophan emission fluorescence upon the addition of acrylamide as a quencher in the range between 0 and 170 mM. For the quenching experiments a 3 μM concentration of peptides was used. Measurements in the presence of liposomes were carried out using a peptide-lipid ratio of 1:1,000 (w/w). Before titration, each peptide was incubated with lipid vesicles for 30 min. Fluorescence spectra were recorded using an excitation wavelength of 295 nm and a scan speed of 200 nm/min. The obtained data were fitted using the Stern–Volmer equations:


$$\frac{F_{0}}{F}=1+K_{sv}\left[Q\right]$$

where $F_{0}$ and *F* are the fluorescence in the absence and in the presence of the acrylamide (*Q*), respectively, and $K_{sv}$ is the Stern–Volmer constant.

Also, the Net Accessibility Factor (NAF) was calculated using the equation:


$$NAF=\frac{K_{sv\;\left(LUV\right)}}{K_{sv\;\left(\text{buffer}\right)}}$$

### 2.5 Membrane permeabilization assay

The uptake of fluorescent probe 1-aminonaphtalene-8-sulfonic acid (ANS) was used for cell permeabilization studies. Specifically, the bacteria used as a model for a Gram− and a Gram+ strain (*Escherichia coli* ATCC 25922, and *Bacillus cereus* ATCC 10876) were grown in Luria Bertani (LB) medium. Subsequently, the cell suspensions were centrifuged at 3600 rpm and 4°C, washed, and resuspended in PBS buffer to achieve an OD_600_ of ~0.6.

Increasing amounts of peptide (ranging from 0 to 20 μM) were added to 700 μL of cell suspension in presence of 25.0 μM of ANS. Fluorescence spectra were recorded from 400 nm to 600 nm, using an excitation wavelength of 360 nm and excitation and emission band-pass of 5 nm and 2.5 nm, respectively. The disruption of cell membrane integrity was quantified by the increase of intensity of fluorescence using the following equation (Della Pelle et al., 2020):


$$\%\text{Uptake}\;ANS=\frac{\left(F-F_{0}\right)}{F}\%$$

where F is the fluorescence of ANS observed at a given peptide concentration and $F_{0}$ is the fluorescence of ANS in the absence of peptides.

### 2.6 Circular dichroism spectroscopy

The secondary structures of the two peptides in a buffer solution (phosphate buffer 10 mM, pH 7.4 and 0.1 mM EDTA) and in presence of the membrane’s mimicking system of POPC and (70/30%) (w/w) POPC/POPG were evaluated by Circular Dichroism (CD) spectroscopy using a J715 JASCO spectropolarimeter.

Briefly, a solution containing 30 μM of peptide was titrated with increasing amounts (ranging between 0 and 2.3 mM) of LUVs. CD spectra were recorded from 190 to 260 nm. The reported spectra are the average of 8 scans with a scanning speed of 100 nm/min, a response time of 2 s, and a bandwidth of 1.0 nm.

The CD signals in millidegrees were converted into mean residue molar ellipticities, ${\left[\theta \right]}_{mr}$ (deg cm^2^ dmol^−1^), using the equation:


$${\left[\theta \right]}_{mr}=\frac{\theta_{Obs}}{10\times C\times l\times \left(N-1\right)}$$

here $\theta_{Obs}$ is the observed ellipticity in millidegrees, *C* is the molar concentration of the peptide, *l* is the path length of the cell in cm, and *N* is the number of amino acids in the peptide.

### 2.7 Bacterial strains

A collection of 50 bacterial strains was used for AMPs susceptibility testing. Those strains are clinical isolates derived from positive blood cultures and each ten strains belong to the following ESKAPE bacterial species: *K. pneumoniae*, *A. baumannii*, *P. aeruginosa*, *E. faecium* and *S. aureus*. Each strain was firstly characterized for genotypic and phenotypic antibiotic-resistance determinants. The genotypic analysis, that also include the species identification, was performed using the FilmArray Blood Culture Identification 2 panel (Bio-Mérieux, Marcy l’Etoile, France). The phenotypic antimicrobial susceptibility testing was performed using VITEK^®^ 2 system with n379 or n397 and xn24 cards (Bio-Mérieux). The susceptibility categorization was performed using EUCAST breakpoint tables version 13.1 (EUCAST, 2014).

### 2.8 Minimum inhibitory concentration (MIC)

To determine the minimum inhibitory concentrations (MIC) of the three antimicrobial peptides, a broth microdilution assay was performed. Bacterial suspensions equal to 0.5 McFarland standard of the individual selected isolates was prepared in saline solution (Fresenius Kabi, Bad Homburg vor der Höhe, Germany) using Densicheck (bio-Mérieux). These suspensions were then diluted in a 1:100 ratio in cation-adjusted Muller Hinton broth (Sigma-Aldrich; St. Louis, Missouri, United States). The final concentration of the peptides ranged from 2 to 128 μg/mL, while the microorganism concentrations were maintained at approximately 5×10^5^ CFU/mL, in compliance with the EUCAST guidelines for the broth microdilution method v5.0 (EUCAST, 2024). In each assay, which was conducted in duplicate, we included both growth controls (microorganism without peptides) and negative controls (peptides without microorganisms). The plates were then incubated overnight at 37°C in an atmosphere of 5% CO_2_ using the New Brunswick™ Excella^®^ E24 Series (New Brunswick Scientific; Edison, New Jersey, United States). The MICs were visually determined as the outcome of this assay. MIC_50_ and MIC_90_ were calculated as previously described (Schwarz et al., 2010).

### 2.9 Minimum bactericidal concentration (MBC)

To determine the Minimum Bactericidal Concentrations (MBC) of the three AMPs, a new broth microdilution assay was conducted, following the same protocol as that employed for MIC determination. After an overnight incubation, for MBC determination, we plated a 3 μL aliquot of each condition present in the 96-well plate onto Mueller Hinton agar plates (Sigma-Aldrich, St. Louis, Missouri, United States). Following an overnight incubation period, we assigned the MBC values to conditions where no visible bacterial growth was observed on the Mueller Hinton agar plates. MBC_50_ and MBC_90_ were calculated as previously described for MIC_50_ and MIC_90_ (Schwarz et al., 2010).

### 2.10 Growth curves

A sensible and resistant representant strain of each species has been randomly selected to perform an optic-based kinetic growth curve obtained using the Cytation5 multimode reader from Biotek (Winooski, Vermont, United States). This reader performed a kinetic protocol spanning 21 h, during which absorbance readings were taken every 30 min in a 96-well plate with a flat bottom (Falcon Corning Incorporated, New York, United States). The experimental setup reflects the broth microdilution assay parameters. The kinetic protocol included plate incubation at 37°C with a 5% CO_2_ atmosphere, accompanied by continuous orbital shaking. The absorbance readings were conducted at a wavelength of 630 nm. The acquired data were subsequently processed and visualized using GraphPad Prism software version 9.3.1 (La Jolla, CA, United States).

### 2.11 Scanning electron microscopy

To evaluate morphological changes (due to the membranolytic activity of the AMPs) on different species tested, a resistant representant strain of each species has been randomly selected. Around 5 × 10^5^ CFU/mL inoculum of each strain has been treated, or not (growth control), for 4 h with peptide concentrations equal to 0.75× MIC_90_ values in cation-adjusted Muller Hinton broth (Sigma-Aldrich). After the treatment, bacterial cells were collected by centrifugation, concentrated in saline solution, and 20 microliters were spread on a sterile Thermanox plastic coverslip (Thermofischer Scientific, Waltham, Massachusetts, United States) and let dry under laminar flow hood.

Afterwards, samples were fixated with 2.5% Glutaraldehyde solution (Sigma-Aldrich) and dehydrated via immersion in crescent gradient of ethanol concentration, from 30 to 100%, with a multi-step procedure of 10 min each. Successively samples were metallized with gold using a High-Resolution Sputter Coater AGB7234 (Agar Scientific, Stansted, United Kingdom). Morphology of cells onto surfaces were observed with Supra25 SEM microscope (Zeiss, Oberkochen, Germany). Representative micrographs were acquired in secondary electrons mode at an acceleration voltage of 8 kV. For each sample at least four randomly selected fields were acquired at magnification of 8,000×.

### 2.12 Hemolytic activity

The peptide’s hemolytic activity was evaluated by testing different concentrations of peptides (from 1.25 to 20 μM, so from 3 to 50 μg/mL) against rabbit erythrocytes (Rockland) previously purified and maintained in Alsever’s solution. After removing Alsever’s solution by centrifugation, the erythrocytes were washed and resuspended in PBS to the appropriate dilution. Following, for each concentration tested, 100 μL of erythrocytes solution (at density of 2.5 × 10^6^ cells/well) were incubated with peptides for 2 h at 37°C. After this time, intact erythrocytes were removed by centrifugation, and the absorbance (A) of the supernatant was measured at 492 nm. Erythrocytes were incubated only with PBS buffer as the negative control and in the presence of Triton X-100 at 2% (v/v) for the positive control. The Hemolysis percentage was calculated as follows (Chen et al., 2023):


$$\%\text{Hemolysis}=\frac{\left(A_{\text{Test}\;\text{group}}-A_{\text{Negative}\;\text{control}}\right)}{\left(A_{\text{Positive}\;\text{control}}-A_{\text{Negative}\;\text{control}}\right)}\%$$

### 2.13 *In vitro* cytotoxicity

The cytotoxicity of the two peptides was determined with the ATPLite Luminescence Assay against a primary human fibroblast cell line (FB789) (Bugli et al., 2022). The cells were grown in Dulbecco’s Modified Eagle Medium (DMEM) containing 10% fetal calf serum (FCS), penicillin, and streptomycin antibiotics at 37°C and in a humidified atmosphere with 5% CO_2_. Briefly, the cells were seeded at a density of 3,000 cells per well in 100 μL of culture medium and were allowed to adhere for 24 h. Subsequently 100 μL of culture medium was removed and replaced with a new culture medium containing the peptide to be tested diluted to the appropriate concentration (from 1.25 to 20 μM, so from 3 to 50 μg/mL). After 3 and 6 h of treatment the cells were lysed and incubated with substrate solution (Luciferase/Luciferin) for 10 min in the dark. The luminescence was measured using a microplate luminometer (Victor II PerkinElmer). As a negative control the cells were grown in medium without the peptides, and for a positive control the cells were grown in medium with 2% v/v NaN_3_. The percentage of cell viability was determined as follows:


$$\%\text{Cells}\;\text{viability}=\frac{{RLU}_{\text{Test}\;\text{group}}}{{RLU}_{\text{Negative}\;\text{control}}}\%$$

where RLU is the unit of relative light measured by the instrument.

### 2.14 *In vivo* toxicity testing on *Galleria mellonella* larvae

The *in vivo* toxicity testing of the peptides was performed using the *Galleria mellonella* larvae as described before (Garcia Maset et al., 2022; Di Vito et al., 2023). The treatments administration was performed with a 0.5 mL syringe into the haemocoel through the last right pro-leg in aseptic conditions. Before injections the pro-leg area was decontaminated with 70% (v/v) Ethanol solution (Carlo Erba, Milan, Italy). Each treatment and control group were composed of 10 larvae. The treatments administered volumes was equal to 10 μL with peptides concentration of 62.5 μg/mL, that is twice the biggest MIC_90_ obtained value (except for Trem-HK and *P. aeruginosa*), 32 μg/mL. The control group was administered with 0.9% NaCl injectable solution (Fresenius Kabi, Bad Homburg vor der Höhe, Germany). After the treatment, larvae were incubated at 33°C in aerobic conditions. The viability of larvae was visually evaluated every 24 h for 72 h; lack of motility after stimulation, and melanization were considered as death indicators according to Loh et al. (2013) criteria. Cocoon formation was excluded as criteria because the phenomenon was not observed in the 3-day observational period.

### 2.15 Statistical analysis

All experiments were repeated at least in triplicate, to ensure reproducibility. Gaussian distribution data were analyzed using mean and standard deviation parameters. *In vivo* toxicity testing on *Galleria mellonella* larvae was performed with 10 replicates per group. Significant difference was defined as *p*-value < 0.05. Numerical data are normally presented in the text as means + SD. Homogeneity of variances was tested before data processing. Data from ATPlite assay were analysed by one-way ANOVA, followed by Bonferroni’s test. Statistical analysis and graphics were performed and obtained using GraphPad Prism software version 9.3.1 (La Jolla, CA, United States).

## 3 Results

### 3.1 Peptide design

Two new peptides, named Trem-HK and Trem-HSK, were designed starting from the sequence of Trematocine, a 22 amino acids AMP identified from the Antarctic fish *Trematomus bernacchii.* This peptide was not cytotoxic and hemolytic and had shown antimicrobial activity against some model bacteria, including *Escherichia coli* and *Bacillus pumilus* (Della Pelle et al., 2020). Since the positive charge is significant for the interaction with the negatively charged bacterial membrane, the sequence of this natural peptide was modified by enhancing the number of positively charged residues. In this way, the peptides affinity for anionic membranes should be improved and, therefore, also their antibacterial activity.

**Figure 1:**
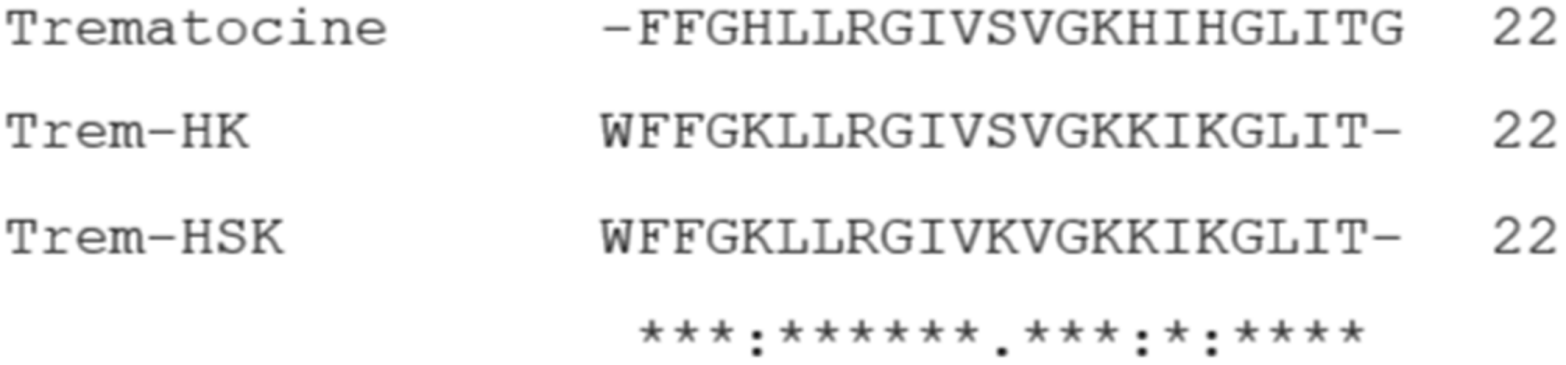
Alignment between Trematocine (Trem), Trem-HK and Trem-HSK peptide sequences. Asterisks (*) indicate amino acid residues that are conserved in all peptides. Colon (:) and dot (.) correspond to positions in which amino acids have been substituted with others showing similar physico-chemical characteristics.

Figure 1 shows an alignment between the peptide sequences of the Trematocine and the two mutants carried out by “Clustal Omega”.^1^

In the Trem-HK peptide, three His residues have been substituted with a Lys, whereas in the Trem-HSK peptide, in addition, a Ser residue has also been changed with a Lys. Moreover, a tryptophan amino acid residue was added at the N-terminus to allow physico-chemical investigations on the peptides by fluorescence spectroscopy, and the final Gly residue was removed to maintain constant the total number of amino acids.

The net charge, molecular weight, isoelectric point and hydrophobic moment of the peptides are reported in Table 1.

**Table 1:** Physico-chemical properties of the trematocine and the two mutant peptides.

Peptide	Net charge	Molecular weight (Da)	pI	
Trematocine	+2	2358.82	11.00	0.550
Trem-HSK	+6	2502.17	11.39	0.488
Trem-HK	+5	2461.08	11.33	0.533

### 3.2 Partition constant determination

Tryptophan fluorescence is sensitive to the environment polarity (Lakowicz, 2006). Thus, tryptophan can be used as probe to study the interaction between peptides and membranes since when tryptophan is in contact with a lipid bilayer, an increase in fluorescence and a blue shift is observed (Freire et al., 2011).

The change of Trp fluorescence was used to evaluate the interaction of Trematocine mutants with two membrane mimetic systems (LUVs), composed of 100% 1-Palmitoyl-2-oleoyl-*sn*-glycero-3-phosphocholine (POPC), that mimic the mammalian cell membranes, and (70/30)% w/w POPC/POPG (1-Palmitoyl-2-oleoyl-*sn*-glycero-3-[phospho-*rac*-(1-glycerol)]), mimicking the bacterial membrane. Supplementary Figure S1 shows the binding isotherms for the peptides upon addition of increasing amount of lipid vesicles.

Table 2 reports the mole fraction partition constant *K_x_*, the free energy of partition, and the selectivity ratio defined as the ratio between the value of the partition constant measured in the presence of POPC/POPG vesicles and the value measured in the presence of POPC vesicles.

**Table 2:** Partition constant, selectivity ratio, and free energy change of partition for the peptides Trem-HK and Trem-HSK in presence of POPC and POPC/POPG LUVs.

Peptide	LUVs	*K_x_*	Selectivity ratio	∆G (kJ/mol)
Trem-HSK	POPC	(4.53 ± 1.02) × 10^5^	1.28	−31.73
	POPC/POPG	(5.80 ± 1.19) × 10^5^		−32.33
Trem-HK	POPC	(5.43 ± 1.12) × 10^5^	1.10	−32.17
	POPC/POPG	(5.98 ± 1.32) × 10^5^		−32.40

The high values of the partition constants indicate that both peptides strongly interact with lipid vesicles partitioning from the water environment. However, the close to 1 value of the selectivity ratio for both peptides indicate a very low selectivity toward one of the two tested membrane models.

### 3.3 Quenching experiments

Fluorescence quenching studies with acrylamide for Trem-HK and Trem-HSK peptides were conducted to further elucidate their interactions with model membranes. We used acrylamide since it is a neutral collisional quencher for Trp and it is a small molecule that can easily diffuse in solution. The obtained quenching values depend on the tryptophan accessibility to the quencher itself (Phillips et al., 1986).

**Figure 2:**
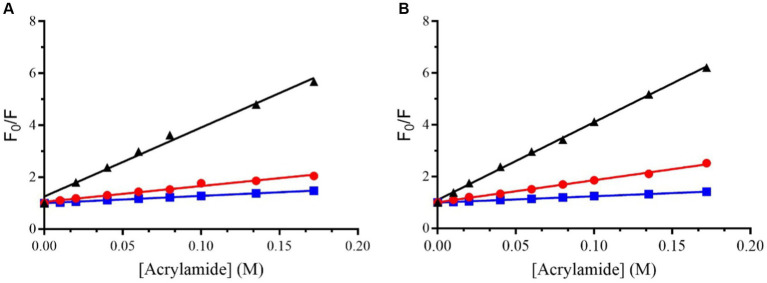
Stern–Volmer plots for the quenching of Trp (the first amino acid of the sequence) in Trem-HSK **(A)** and Trem-HK **(B)** measured in buffer (black), in the presence of POPC (red) and in the presence of (70/30)% w/w POPC/POPG (blue).

Figure 2 shows the classical Stern-Volmer plots obtained for Trem-HSK and Trem-HK peptides in buffer and in the presence of LUVs of different composition.

Table 3 shows the values of the Stern-Volmer constants (K_SV_), obtained by the Stern-Volmer equation, and the NAF (Net Accessibility Factor). The reciprocal of K_SV_ is the quencher concentration capable of decreasing the initial fluorescence by 50%. Therefore, the value of the Stern-Volmer constant is indicative of the exposure of the fluorophore (Trp) to the quencher and to the solvent. The lower values obtained in the presence of liposomes, compared to the value measured in buffer, indicate a decrease in quenching efficiency resulting from a lower exposure of tryptophan to the solvent. The results obtained from the partition measurements and the quenching experiments show that both peptides interact with the liposomes formed by POPC and POPC/POPG.

**Table 3:** Stern-Volmer constants and NAF values for the two peptides for the different tested conditions.

Peptide	System	K_sv_ (M^−1^)	NAF
Trem-HSK	Buffer POPC POPC-POPG	27.0 ± 0.7 6.0 ± 0.1 3.00 ± 0.02	1.00 0.22 0.11
Trem-HK	Buffer POPC POPC-POPG	27.0 ± 0.9 9.0 ± 0.3 2.40 ± 0.03	1.00 0.33 0.09

The NAF value, lower in the presence of POPC/POPG than in the presence of POPC for both peptides, indicates a more robust interaction with anionic membrane models, making the Trp less accessible to the quencher.

### 3.4 ANS membrane permeabilization assay

To determine the ability of Trem-HK and Trem-HSK peptides to disrupt and permeabilize the outer membrane of a model Gram-negative bacteria (*Escherichia coli* ATCC 25922) and the plasmatic membrane of a model Gram-positive bacteria (*Bacillus cereus* ATCC 10876) we carried out the permeabilization assay using the fluorescent probe (ANS).

The fluorescence of ANS is relatively weak in an aqueous solution and strong in hydrophobic environment. ANS is not able to pass the bacterial membrane. Still, upon addition of peptides capable of interfering with the cell membrane integrity, it can be incorporated into the lipid bilayer and, therefore, its fluorescence intensity increases drastically (Schäfer and Wenzel, 2020).

**Figure 3:**
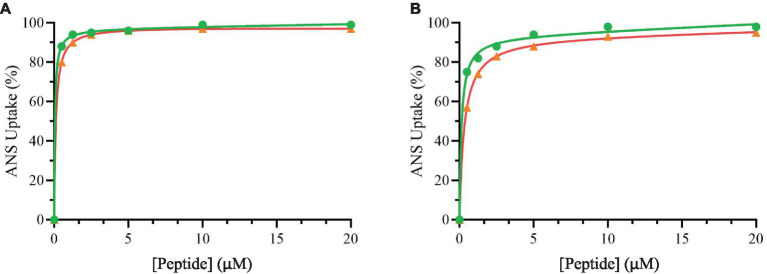
Percentage of ANS uptake of *Bacillus cereus* (orange), *Escherichia coli* (green) as a function of peptide Trem-HSK **(A)** and Trem-HK **(B)** concentration.

Figure 3 shows the percentage of ANS uptake at the different tested concentrations of peptides.

From these data it is possible to highlight that the peptides have a great ability to alter both the plasmatic membrane of *Bacillus cereus* and the outer membrane of *Escherichia coli*. In fact, the percentage of uptake is already very high at low concentration values.

### 3.5 Circular dichroism spectroscopy

**Figure 4:**
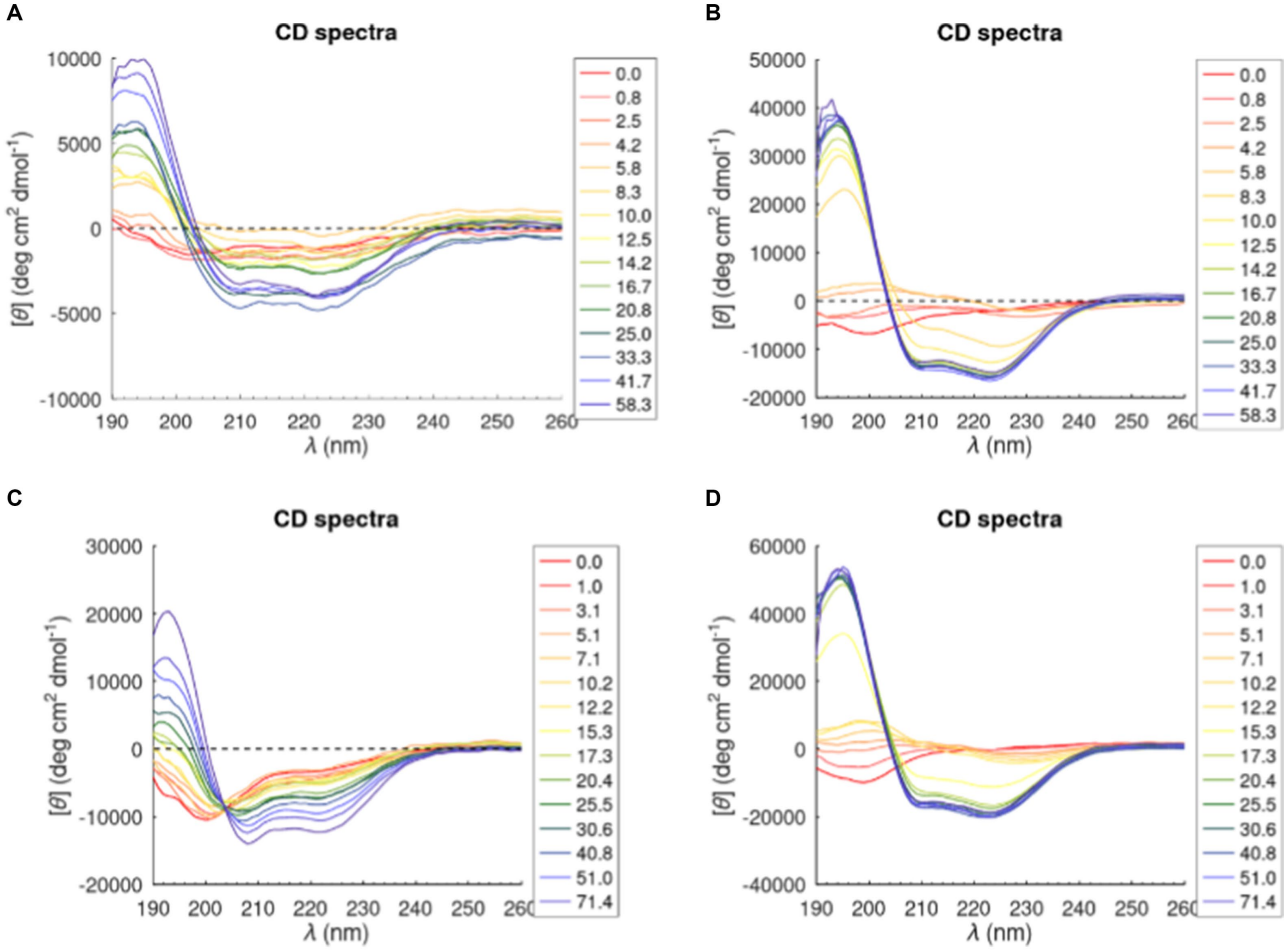
CD spectra of Trem-HK and Trem-HSK peptides in POPC **(A)** and **(C)** and (70/30)% w/w POPC—POPG **(B)** and **(D)**. The legend shows the lipid/peptide ratio value in each graph.

Most antimicrobial peptides are unstructured in aqueous solutions, but when they interact with membranes a total or partial transition into an α-helical structure can be observed. CD studies were performed to investigate the structural changes of the two peptides in the presence of the tested model membrane. CD spectra are reported in Figure 4. A strong negative band at ~200 nm (red line) characteristics of random coil conformation was highlighted in buffer. Upon addition of lipid vesicles, a positive band at ~195 nm and two minima at ~208 nm and ~ 222 nm show up, suggesting that the peptides were assuming an α-helical conformation.

The percentage of α-helical content of Trem-HK and Trem-HSK in presence of POPC and POPC-POPG vesicles was calculated from experimental CD data by using the K2D3 algorithm (Louis-Jeune et al., 2012) (Supplementary Figure S2). The percentage of α-helical content, for both peptides, increases with the increase of lipid-peptide ([L]/[P]) ratio passing from zero (random coil, [L]/[P] = 0) to ~75% in the presence of POPC-POPG vesicles and 30–40% in presence of POPC vesicles for a [L]/[P] ratio above of 16.7. Moreover, Trem-HK and Trem-HSK show a higher α-helix content, for each [L]/[P] ratio, in the presence of anionic membrane (POPC-POPG) with respect to zwitterionic membrane (POPC) (Louis-Jeune et al., 2012).

### 3.6 Clinical isolates characterization

The bacterial collection consists of clinical strains isolated from monomicrobic positive blood cultures, and successively tested genotypically and phenotypically for species identification and AMR characterization. The search for AMR traits included qPCR-based detection of some important resistance-associated genes from primary sample and antimicrobial susceptibility testing from sub-cultured plates.

As shown in Table 4, the collection divided each species in five Resistant (R) and five Susceptible (S) strains based on the upcoming criteria. *K. pneumoniae* and *P. aeruginosa* resistant isolates involved in this study must be intended as MDR strains alongside with ESBL genotype and phenotype involving carbapenem resistance (CRE and CRPA, respectively). *A. baumannii* resistant strains, are carbapenem-resistant *A. baumannii* (CRAB) with an additional Colistin resistance to discriminate them from the sensible clinical isolate, this classification was made on AST phenotypic base (*bla*_OXA-23_ is not a target in the used syndromic panel). Gram-positive bacteria *S. aureus* and *E. faecium* are indicated as resistant strains to Methicillin (MRSA) and Vancomycin (VRE) respectively, based on genetic presence of resistance genes, but also confirmed by Oxacillin and Vancomycin MIC values.

**Table 4:** Summarizing table of the bacterial collection used in this study.

Species	Strain number (R/S)	Antimicrobial resistance genes detected
*Enterococcus faecium*	10 (5/5)	*van*_A_ (4), *van*_B_ (1)
*Staphylococcus aureus*	10 (5/5)	*mec*_A_ (3), *mec*_C_ (2)
*Klebsiella pneumoniae*	10 (5/5)	*bla*_KPC_ (3*), *bla*_KPC_ & *bla*_OXA-48_ (1), *bla*_NDM_ & *bla*_CTX-M_ (1)
*Acinetobacter baumannii*	10 (5/5)	–
*Pseudomonas aeruginosa*	10 (5/5)	*bla*_VIM_ (2), *bla*_IMP_ (2), *bla*_GES_ (1)

The table stratifies the bacterial features regarding species, strain number and categorization (R/S), and antimicrobial resistance genes detected. *One of the bla_KPC_ positive *K. pneumoniae* results as sensible to carbapenem drugs and resistant to the β-Lactam-β-Lactamase inhibitor combination Ceftazidime-avibactam (CAZ-AVI). This behavior is often observed in mutated bla_KPC-14_ or bla_KPC-3_ strains, reflecting amino acid deletions or substitutions that affect both carbapenem and CAZ-AVI susceptibility (Shields et al., 2017; Niu et al., 2020).

### 3.7 Minimum inhibitory and bactericidal concentrations

The two peptides were strategically designed to increase their positive charges to enhance their interaction with bacterial membranes, thereby boosting their antibacterial efficacy. The obtained results do not demonstrate any difference in susceptibility between the resistant and susceptible subgroups of each species. Notably, as evidenced in Table 5, the Minimum Inhibitory Concentration values for three of five bacterial species have at least halved compared to those observed with the wild-type peptide. Particularly noteworthy is the significant reduction for *Acinetobacter baumannii* mean MIC, which shows a substantial drop from a value bigger than 32 μg/mL (13.57 μM) to just 8 and 7.6 μg/mL (3.39 and 3.22 μM) for both Trem-HK and Trem-HSK. In general, the results consistently demonstrate a reduction in MIC values or, at most, the maintenance of similar values. Moreover, regarding the MBC results, it is possible to highlight an improvement of bactericidal activity due to the peptide mutations. As an example, mean MBC values related to *A. baumannii* decreased from a value bigger than 32 μg/mL (13.57 μM) to 8.8 μg/mL (3.73 μM) for both mutants. The mutant peptides’ MIC and MBC were significantly decreased for the 20 isolates of the gram-positive bacteria: *Enterococcus faecalis* seems to be the most effected by the charge-positivization mutations of the Trematocine.

**Table 5:** Minimum Inhibitory (MIC) and Bactericidal (MBC) mean concentrations of the tested ESKAPE bacteria (*n* = 50) divided by species (10 strains each).

	MIC ± SD (μg/mL)			MIC_50_ (μg/mL)			MIC_90_ (μg/mL)		
	Trem WT	Trem HK	Trem HSK	Trem WT	Trem HK	Trem HSK	Trem WT	Trem HK	Trem HSK
*E. faecium* (n = 10)	23.2 ± 9.6	6.8 ± 1.9	6.4 ± 2.1	32	8	8	32	8	8
*S. aureus* (n = 10)	15.2 ± 2.5	7.6 ± 1.3	8.0 ± 0.0	16	8	8	16	8	8
*K. pneumoniae* (n = 10)	>32	32.0 ± 0.0	30.4 ± 5.1	>32	32	32	>32	32	32
*A. baumannii* (n = 10)	>32	8.0 ± 0.0	7.6 ± 1.3	>32	8	8	>32	8	8
*P. aeruginosa* (n = 10)	>32	>32	28.8 ± 6.7	>32	>32	32	>32	>32	32

	MBC ± SD (μg/mL)			MBC_50_ (μg/mL)			MBC_90_ (μg/mL)		
	Trem WT	Trem HK	Trem HSK	Trem WT	Trem HK	Trem HSK	Trem WT	Trem HK	Trem HSK
*E. faecium* (n = 10)	30.4 ± 5.1	8.8 ± 2.5	7.6 ± 1.3	32	8	8	32	8	8
*S. aureus* (n = 10)	23.2 ± 9.6	9.2 ± 3.8	8.8 ± 2.5	32	8	8	32	8	8
*K. pneumoniae* (n = 10)	>32	>32	32.0 ± 0.0	>32	>32	32	>32	>32	32
*A. baumannii* (n = 10)	>32	8.8 ± 2.5	8.8 ± 2.5	>32	8	8	>32	8	8
*P. aeruginosa* (n = 10)	>32	>32	>32	>32	>32	>32	>32	>32	>32

The table also comprehend MIC_50_, MBC_50_, MIC_90_ and MBC_90_ values. The concentrations are expressed in μg/mL unit.

Moreover, in most cases the two mutants show similar values of both MIC and MBC for the tested strains: the inhibitory and bactericidal activity of the peptides seem to be strictly related. The antimicrobial susceptibility testing data for both common antibiotic and antimicrobial peptides studied is available as a database (Supplementary material S1).

### 3.8 Bacterial growth curves

**Figure 5:**
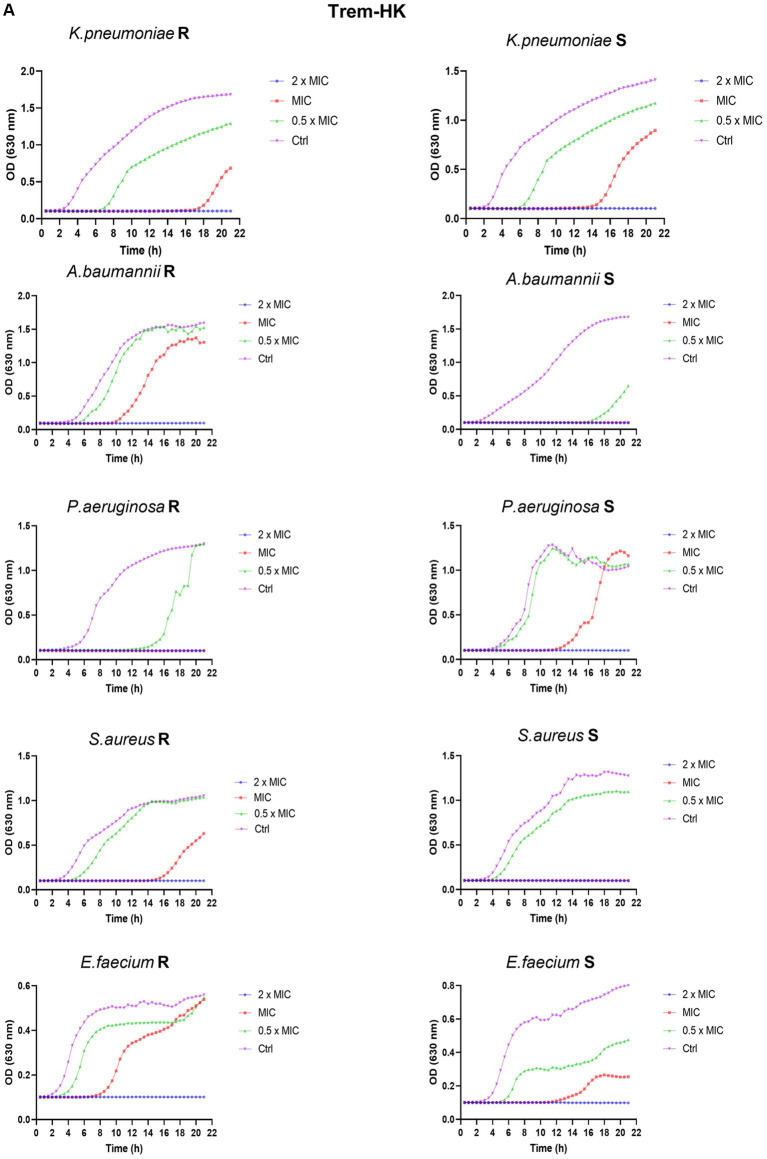

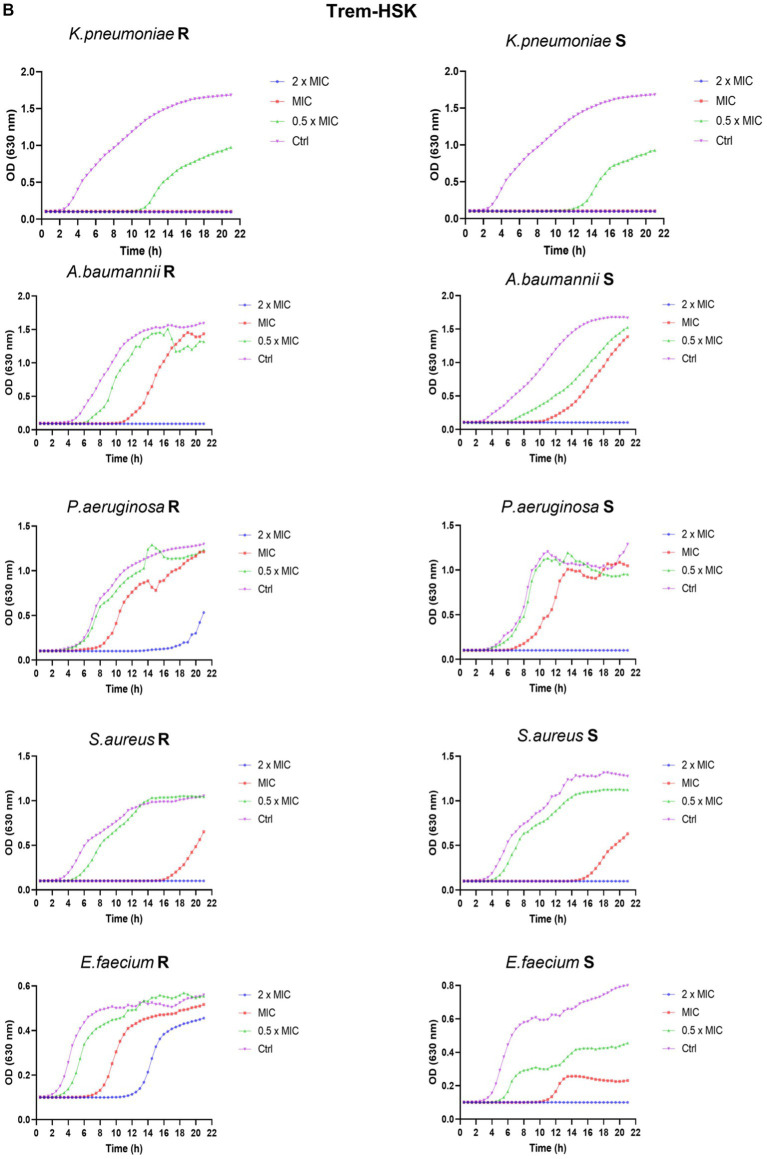
Panel **(A,B)** reports the growth curves of Trem-HK and Trem-HSK for the different tested bacterial strains. R = resistant. S = susceptible (following categorization stated in Section 3.6).

To thoroughly investigate the antimicrobial properties of mutant peptides, our study explored their effects on microbial growth within a kinetic setting. As a result, we produced growth curves for a sensible and resistant representants, randomly chosen, of each ESKAPE species exposed to concentrations of the two mutated peptides at 0.5 × MIC_90_, MIC_90_, and 2 × MIC_90_. For MIC_90_ values above 32 μg/mL, the tested concentrations are 128, 64 and 32 μg/mL (indicated in Figure 5 as 2 × MIC, MIC and 0.5 × MIC respectively). During a 21-h period, we followed changes in optical density (O.D.) to gain valuable insights into growth kinetics. In all kinetics experiments, when using twice the MIC_90_ concentrations of both peptides, microbial growth is completely inhibited. However, when using the MIC_90_ concentration, we observe a delay in the rise of the growth curves, along with lower absorbance readings during the plateau phase. The growth curves consistently illustrate a dose-dependent inhibition of microbial growth. In comparison, the untreated growth control for various bacterial isolates, whether susceptible or resistant, exhibits the highest optical density (OD) values and enters the exponential growth phase earlier than the samples treated with different concentrations of antimicrobial peptides. These comparative growth curves (Figures 5A,B) provide strong evidence of the rapid and effective cytocidal action of the two peptides.

### 3.9 Scanning electron microscopy

**Figure 6:**
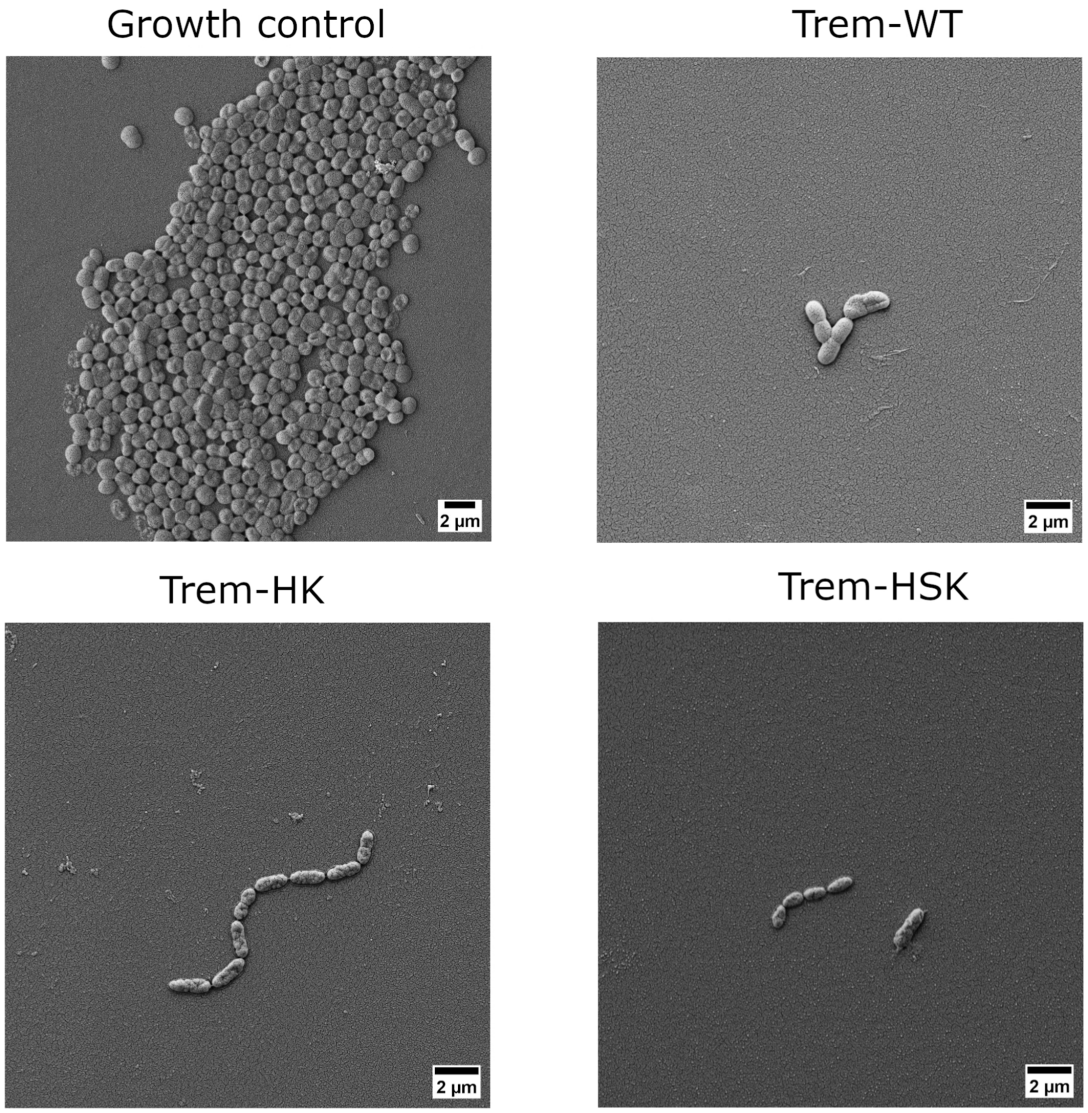
Scanning electron micrographs of a colistin-resistant *A. baumannii* cells untreated or treated with 0.75 × MIC of Trem-WT, Trem-HK and Trem-HSK for 4 h. Magnification is 8,000× and scale bar is equal to 2 μm.

**Figure 7:**
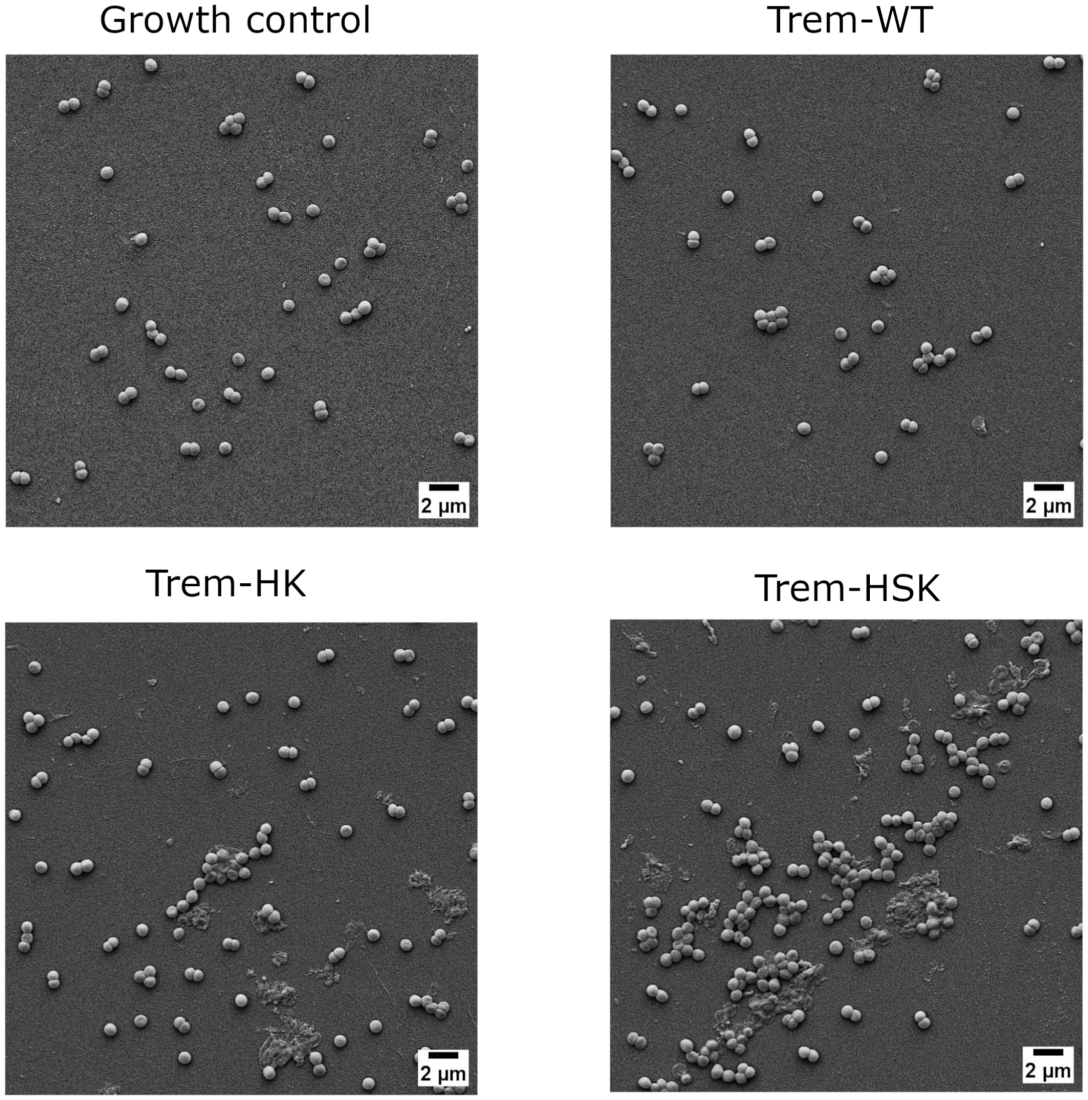
Scanning electron micrographs of Methicillin-resistant *S. aureus* strain cells untreated or treated with 0.75 × MIC of Trem-WT, Trem-HK and Trem-HSK for 4 h. Magnification is 8,000× and scale bar is equal to 2 μm.

The obtained data from biochemical and antimicrobial characterizations suggest that Trematocine mutants have an enhanced membranolytic activity against most of the ESKAPE species. Gram-positive species seems to be the more effected by Trematocine itself, while aminoacidic mutations in addition to ameliorate bactericidal potency, broaden the antibacterial activity at low concentrations for *A. baumannii*. To better investigate the improved membranolytic action of mutans, a high magnification microscopy has been performed. Scanning electron imaging was utilized after a short-term treatment of 4 h with 0.75× MIC_90_ concentrations of Trematocine and mutants, with a gram-negative and a gram-positive representant of ESKAPE opportunistic pathogens: Colistin-resistant *A. baumannii* and Methicillin-resistant *S. aureus, respectively.* For MIC_90_ values above than 32 μg/mL, the tested concentration (compared with 0.75 × MIC) is 32 μg/mL. The magnification used (Figures 6, 7) to assess the presence of membranolytic activity on *A. baumannii* and *S. aureus* is 20,000×, while micrographs of *K. pneumoniae*, *P. aeruginosa*, and *E. faecium* were acquired at 8,000× to 20,000× magnification (Supplementary Figures S3–S5 respectively).

As shown in Figure 6, *A. baumannii* growth control cells have an approximal length of 1.5 μm, some evident bacterial division secta and a swollen appearance. In contrast Trem-HK and Trem-HSK seems to produce a cell wall and membrane alteration of the gram-negative bacteria, evident from the shrunken appearance and for the reduced perpendicular extension to the main axis of the bacillus. Trem-WT, differently from the double and triple mutants of Trematocine, have a less extent impact on the bacterial morphology and membrane integrity: the majority of cells in the micrograph has a morphological integrity similar to growth control, and only one cell exhibits deflated and potentially symptom of an enhanced bactericidal activity.

Figure 7 display the SEM imaging session for a Methicillin-resistant *S. aureus* strain involved in the study. Staphylococcal cells in the growth control appeared grouped and isotonic, while the Trematocine-treated one seems suffering and undersized. Trematocine and both mutants, HK and HSK, provoke an extended morphological alteration to cell wall and membrane with and evident shrinking and elongation of cocci.

### 3.10 Hemolytic activity

**Figure 8:**
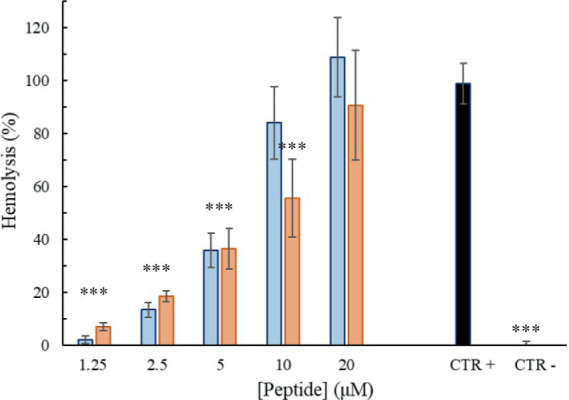
Percentage of hemolysis as a function of peptide Trem-HSK (orange) and Trem-HK (blue) concentration. The asterisks indicate the significance level respect to positive control (black): ***p < 0.001.

To get more information of the Trem-HK and Trem-HSK peptides selectivity toward bacterial cells, their hemolytic activity against rabbit red blood cells was investigated. Overall, the peptides displayed little to no hemolysis of erythrocytes until 2.5 μM (6.1 μg/mL), with values of about 35% at 5 μM (12.2 μg/mL) as shown in Figure 8. At higher concentrations, the peptides caused up to 80–100% hemolysis.

### 3.11 Cytotoxic activity

**Figure 9:**
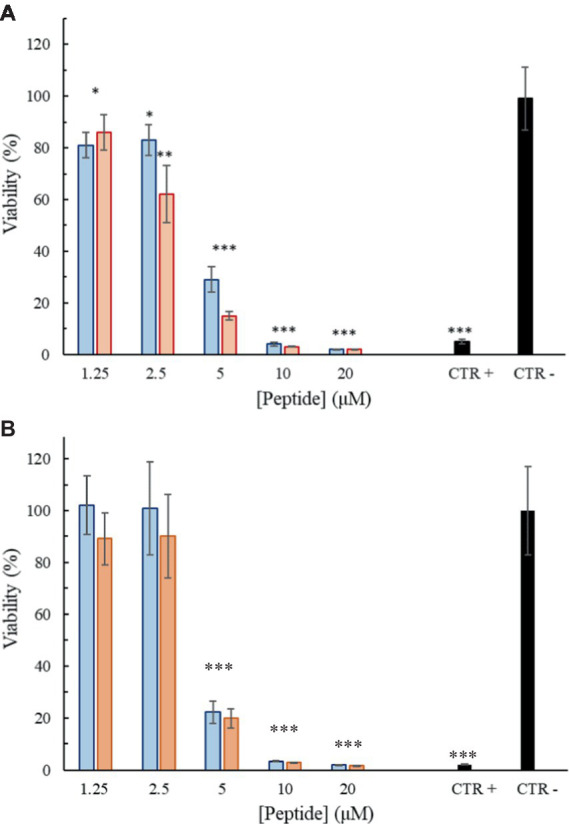
Percentage of viability as a function of peptide Trem-HSK (red) and Trem-HK (blue) concentration after 3 **(A)** and 6 **(B)** hours of treatment. The asterisks indicate the significance level respect to negative control (black): *p < 0.05; **p < 0.01; ***p < 0.001.

To investigate the effect of the peptides on the viability of mammalian cell lines, an ATP-lite assay was performed using the primary fibroblast cell line FB-789. Cell vitality was evaluated after 3 and 6 h of treatment with different concentrations of the peptides. As shown in Figures 9A,B, the peptides were little or no cytotoxic until 2.5 μM (6.1 μg/mL) at the two considered time points. However, already at a concentration of 5 μM (12.2 μg/mL) a significant reduction in the cell viability can be observed in comparison to the negative control (100% of viability). Trem-HK seems less cytotoxic than Trem-HSK.

### 3.12 Assessment of *in vivo* toxicity of Trem-HK and Trem-HSK using a *Galleria mellonella* larvae model

**Figure 10:**
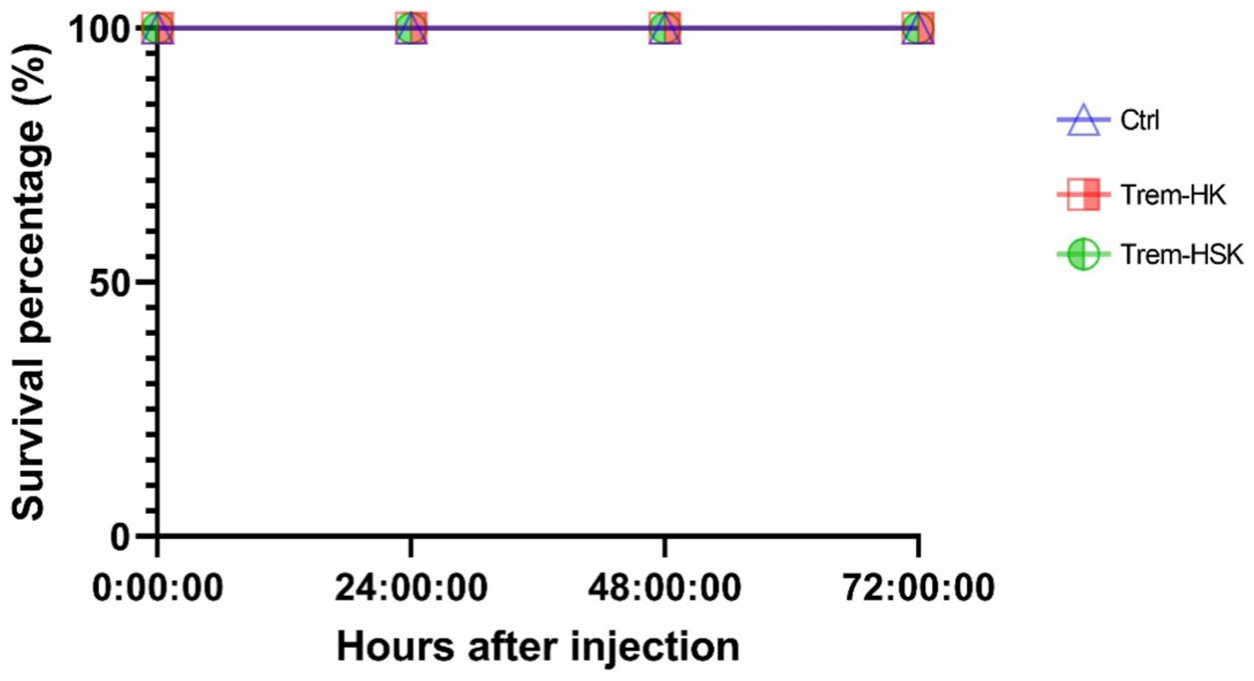
Toxicity of trematocine mutants on an insect larvae model.

To assess the toxicity of Trem-HK and Trem-HSK, we conducted *in vivo* experiments using *Galleria mellonella* larvae as a model organism. A single concentration of 62.5 μg/mL (25.5 μM) was selected for the toxicity test on the larvae. It’s worth noting that this concentration, for example, is about eight times higher than the lowest MIC_90_ value obtained for *A. baumanni*. Following the administration of both peptides, we monitored the larvae for a period of 72 h, looking for any signs of toxicity such as melanization or reduced motility. Remarkably, as shown in Figure 10, no signs of toxicity were observed in any of the treated larvae when compared to the control group, and all larvae maintained 100% viability throughout the experiment. These results demonstrate an outstanding biocompatibility profile for both mutant peptides, suggesting their good potential for application in clinical settings. The discrepancy between *in vitro* and *in vivo* toxicity evaluations can be reflected by intrinsic differences in study models and pharmacokinetic variables.

## 4 Discussion

Antimicrobial resistance (AMR) stands as a grave public health concern, particularly in the context of bacterial infections. The escalating impact of antibiotic-resistant strains has led to a significant global health crisis. The WHO has identified the ESKAPE group—*Enterococcus faecium*, *Staphylococcus aureus*, *Klebsiella pneumoniae*, *Acinetobacter baumannii*, *Pseudomonas aeruginosa*, and *Enterobacteriaceae* family.—as urgent targets due to their exceptional ability to evade conventional antibiotics (De Oliveira et al., 2020; Abebe and Birhanu, 2023). Addressing the critical need for novel strategies against resistant infections and the development of new antimicrobials is, therefore, fundamental. AMPs emerge as promising candidates, offering a potential breakthrough in facing AMR challenges. With their ability to target a broad spectrum of pathogens, including the ESKAPE group, through interactions with cell membranes, AMPs present an attractive alternative for innovative antimicrobial treatments (Bugli et al., 2022; Ji et al., 2024; Straus, 2024). Their potential to minimize the appearance of resistance further enhances their appeal. In this research, we explored the biological activity of two mutants derived from the scaffold of trematocine, an AMP identified in the red-blooded Antarctic fish *Trematomus bernacchii* (Della Pelle et al., 2020). The site-specific mutations were strategically designed to enhance the bactericidal activity of the native peptide, primarily achieved by augmenting the positive charge and facilitating a greater affinity of the peptides for the bacterial cellular membranes (Dathe et al., 2001). Different papers have emphasized the important impact of positive charges in the electrostatic interactions between AMPs and membranes, studying their mode of insertion and ability to alter bilayer properties (Alfred et al., 2024). Therefore, Trem-HK and Trem-HSK have been designed to have a net positive charge increase, compared to the natural peptide, from +2 to +5 and + 2 to +6, respectively. We studied the effects of these mutations, evaluating the ability of peptides to interact with different membrane-mimicking systems. Partition constant values for LUVs mimicking mammalian and bacterial membranes are 10 time higher compared to trematocine (Della Pelle et al., 2020) for the two mutants, but there is no indication of a selectivity for a specific cell wall. Stern-Volmer constants indicates, for both mutant peptides, that the Trp at the N-terminus get inserted in the interfacial region of the lipid bilayer in presence of LUVs, whereas the NAF values are lower for POPC-POPG membranes, indicating a lower accessibility of Trp-1 to the quencher in this case. The ability of Trem-HK and Trem-HSK peptides to permeabilize the outer membrane of *E. coli* was greatly improved compared to the trematocine and, therefore, an effect due to the increase of the net positive charge was highlighted. Finally, both peptides assume an α-helical conformation in the presence of LUVs, with a higher percentage of secondary structure evidenced in the presence of increasing concentrations of anionic membrane.

Trem-HK and Trem-HSK, moreover, present compelling evidence of their enhanced antibacterial efficacy at the expense of a slightly lower biocompatibility profile. The antimicrobial evaluation was performed on an important collection of MDR and susceptible strains of the WHO’s prioritized list of bacterial pathogens, the ESKAPE group. As described in Table A2.1 of the WHO Bacterial Priority Pathogens List (WHO, 2024), the ESKAPE bacteria selected in this study are all included in medium–high (21–30%) or high (> 30%) mortality rate list. To better summarize the intrinsic heterogeneity of these resistant bacteria, each species has at least two different AMR associated genes variants detected. The bacterial collection presents the most represented genotypes detected in recent surveillance studies and systematic analyses published on antibiotic-resistant bacterial threats in public health (Jean et al., 2022; Mestrovic et al., 2022; WHO, 2024).

The observed reduction in MIC values across various bacterial species, especially the substantial drop for *A. baumannii* and *E. faecium*, is a noteworthy achievement. The designed positive charge strategy appears effective, considering this parameter, in enhancing the peptides’ interaction with bacterial membranes. The consistency in the reduction or maintenance of MIC values implies a broad-spectrum antibacterial potential. Additionally, the improvements in MBC values, particularly for *A. baumannii* and *E. faecium*, underscore the peptides’ enhanced bactericidal activity, supporting their potential therapeutic utility. The growth curve experiments provide valuable insights into the kinetics of peptides microbial inhibition. The dose-dependent inhibition of microbial growth, illustrated by delayed rise and lower absorbance readings, is a strong indicator of the peptides’ effectiveness. The complete inhibition at 2 × MIC_90_ emphasizes their potency. The comparison with untreated controls further supports the peptides’ antimicrobial efficacy, as evidenced by the rapid and effective cytocidal action highlighted by growth-rate differences in the curves.

The morphological characterization of the bactericidal effect of the tested peptides on ESKAPE representants was conducted with a slightly reduced peptide concentration (0.75 × MIC) to avoid extended bacterial lysis during the 4-h treatment and to focus on cell wall and membrane physiology alterations. The scanning electron microscopy was performed till 20,000× magnification to have enough resolution to investigate superficial alterations of bacterial cells when compared to control samples, and to include more than one cell per micrograph, when applicable. Both CRAB and VRE isolates, included as critical and high priority AMR pathogens by WHO (2024), show an enhanced cell wall and membrane alterations when treated with Trematocine mutants, compared to wild-type peptide. The differences are even more evident for Trem-WT, when bacterial integrity and numerosity is compared to control samples. The membranolytic activity of Trematocine and its mutants, already demonstrated for reference model of bacterial strains, is also confirmed by high-magnification microscopy on clinical multi-drug resistant bacteria. Cell wall and membrane differences between gram-negative and gram-positive bacteria seem to have no impact on Trem-HK and Trem-HSK peptides, while *Pseudomonas aeruginosa* isolates show a better resilience to those treatments.

The investigation of hemolytic activity against rabbit red blood cells indicates low hemolysis until 2.5 μM (6.1 μg/mL), suggesting a favorable selectivity toward bacterial cells. However, the increase in hemolysis at higher concentrations raises concerns about potential toxicity. Optimizing antimicrobial peptides requires a delicate balance between maximizing antimicrobial efficacy and ensuring a favorable safety profile. While enhancing binding affinity with biological membranes may result in a modest increase in cytotoxicity, our study observed only a marginal increment in this regard. Overall, further exploration and optimization of peptide concentrations may be necessary to minimize adverse effects while maintaining antibacterial efficacy. The cytotoxicity assessment on mammalian cell lines reveals a non-cytotoxic effect until 2.5 μM (6.1 μg/mL), aligning with the favorable selectivity observed in hemolytic activity. As discussed for peptides hemolytic activity, the significant reduction in cell viability at 5 μM (12.2 μg/mL) opens questions about the peptides’ potential cytotoxicity at higher concentrations. The cytotoxicity assessment on mammalian cell lines reveals a non-cytotoxic effect until 2.5 μM (6.1 μg/mL), aligning with the favorable selectivity observed in hemolytic activity. As discussed for peptides hemolytic activity, the significant reduction in cell viability at 5 μM (12.2 μg/mL) opens questions about the peptides’ potential cytotoxicity at higher concentrations. The use of the *Galleria mellonella* model to assess *in vivo* toxicity is a valuable step towards understanding the peptides’ safety profile. The absence of toxicity signs in treated larvae, even at a concentration eight times higher than the lowest MIC for *A. baumannii*, is promising. The 100% viability throughout the experiment suggests a high level of biocompatibility, supporting the peptides’ potential for clinical applications. This result underscores the superiority of *in vivo* testing in providing a more reliable understanding of the peptides’ safety profile and it validates the peptides’ viability for practical medical applications emphasizing the importance of filling the gap between *in vitro* and *in vivo* assessments for a more robust evaluation of safeness.

In conclusion, the rationale design of these mutants has added new insight on the impact of adding charge residue on the biological activity of a natural AMP, evidencing that the increase improves antimicrobial properties of peptides but also collide with their safety profile.

## Data availability statement

The original contributions presented in the study are included in the article/Supplementary material, further inquiries can be directed to the corresponding author.

## Ethics statement

Ethical approval was not required for the studies on animals in accordance with the local legislation and institutional requirements because only commercially available established cell lines were used. Directive 2010/63/EU of the European Parliament and Council of 22 September 2010 on the protection of animals used for scientific purposes does not include invertebrates as an object of ethical regulation.

## Author contributions

DS: Conceptualization, Data curation, Formal analysis, Funding acquisition, Investigation, Methodology, Project administration, Resources, Software, Supervision, Validation, Visualization, Writing – review & editing. FM: Conceptualization, Data curation, Formal analysis, Funding acquisition, Investigation, Methodology, Project administration, Resources, Software, Supervision, Validation, Visualization, Writing – review & editing. MG: Conceptualization, Data curation, Formal analysis, Funding acquisition, Investigation, Methodology, Project administration, Resources, Software, Supervision, Validation, Visualization, Writing – review & editing. SB: Conceptualization, Data curation, Formal analysis, Funding acquisition, Investigation, Methodology, Project administration, Resources, Software, Supervision, Validation, Visualization, Writing – review & editing. MC: Writing – original draft, Writing – review & editing. MV: Writing – original draft, Writing – review & editing. FP: Conceptualization, Data curation, Formal analysis, Funding acquisition, Investigation, Methodology, Project administration, Resources, Software, Supervision, Validation, Visualization, Writing – review & editing. RR: Conceptualization, Data curation, Formal analysis, Funding acquisition, Investigation, Methodology, Project administration, Resources, Software, Supervision, Validation, Visualization, Writing – review & editing. FC: Conceptualization, Data curation, Formal analysis, Funding acquisition, Investigation, Methodology, Project administration, Resources, Software, Supervision, Validation, Visualization, Writing – review & editing. MS: Writing – original draft, Writing – review & editing. FBuo: Conceptualization, Data curation, Formal analysis, Funding acquisition, Investigation, Methodology, Project administration, Resources, Software, Supervision, Validation, Visualization, Writing – original draft, Writing – review & editing. FBug: Conceptualization, Data curation, Formal analysis, Funding acquisition, Investigation, Methodology, Project administration, Resources, Software, Supervision, Validation, Visualization, Writing – original draft, Writing – review & editing.

## Footnotes

^1^ https://www.ebi.ac.uk/Tools/msa/clustalo
