# Supplementary material for: Trematocine-derived antimicrobial peptides from the Antarctic fish Trematomus bernacchaii: potent antibacterial agents against ESKAPE pathogens
Source: Front Microbiol. 2024 Aug 7;15:1447301. doi: 10.3389/fmicb.2024.1447301 (PMC11335685; doi:10.3389/fmicb.2024.1447301)
Supplement: Supplementary file 2 [file Table_1.DOCX]

**Trematocine-derived antimicrobial peptides from the Antarctic fish *Trematomus bernacchii*: potent antibacterial agents against ESKAPE pathogens**

**Damiano Squitieri^1†^, Federica Massaro^2†^, Monica Mollica Graziano^2^, Stefano Borocci^2,3^, Margherita Cacaci^1,4^, Maura Di Vito^1^, Fernando Porcelli^2^, Roberto Rosato^1^, Francesca Ceccacci^3^, Maurizio Sanguinetti^1,4#^ Francesco Buonocore^2#^, Francesca Bugli^1,4#^**

^1^Dept. of Basic Biotechnological Sciences, Intensive and Perioperative Clinics, Catholic University of the Sacred Heart, Rome, 00168, Italy.

^2^Dept. for Innovation in Biological, Agro-food and Forest systems (DIBAF), University of Tuscia, Largo dell'Università snc, 01100 Viterbo, Italy.

^3^Institute for Biological Systems of Italian National Research Council (ISB-CNR), Secondary Office of Rome-Reaction Mechanisms c/o Department of Chemistry, La Sapienza University of Rome, Piazzale Aldo Moro 5, 00185 Rome, Italy

^4^Dept. of Laboratory Sciences and Infectious Diseases, A. Gemelli University Hospital Foundation IRCCS, 00168 Rome, Italy.

**^†These authors contributed equally to this work and share first authorship^**

**^# These authors share last authorship^**

*** Correspondence:**
Francesco Buonocore, Dept. for Innovation in Biological, Agro-food and Forest systems (DIBAF), University of Tuscia, Largo dell'Università snc, 01100 Viterbo, Italy,Tel. 0039 761 357644; fbuono@unitus.it.

Francesca Bugli, Dept. of Basic Biotechnological Sciences, Intensive and Perioperative Clinics, Catholic University of the Sacred Heart, Rome, 00168, Italy, Tel. 00390630154964; [francesca.bugli@unicatt.it](mailto:francesca.bugli@unicatt.it)

Keywords: Antimicrobial peptides (AMP); Antimicrobial resistance (AMR); ESKAPE pathogens; Membranolytic agents; Multi-drug resistant (MDR) bacteria.

(Min.5-Max. 8)

1. *B)*

*
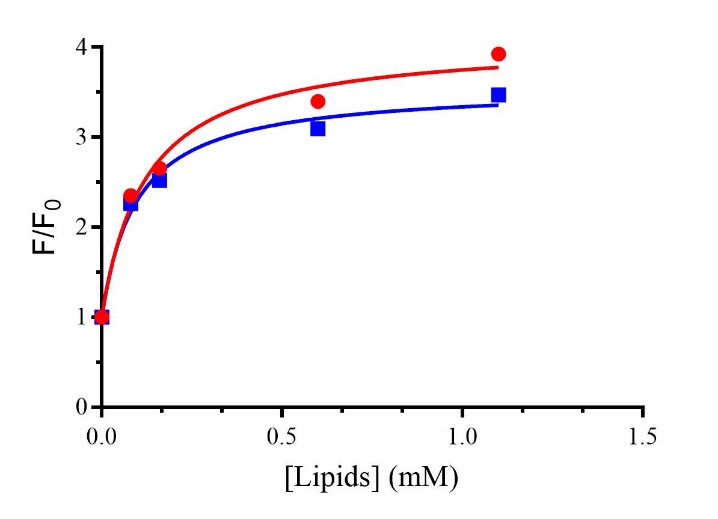

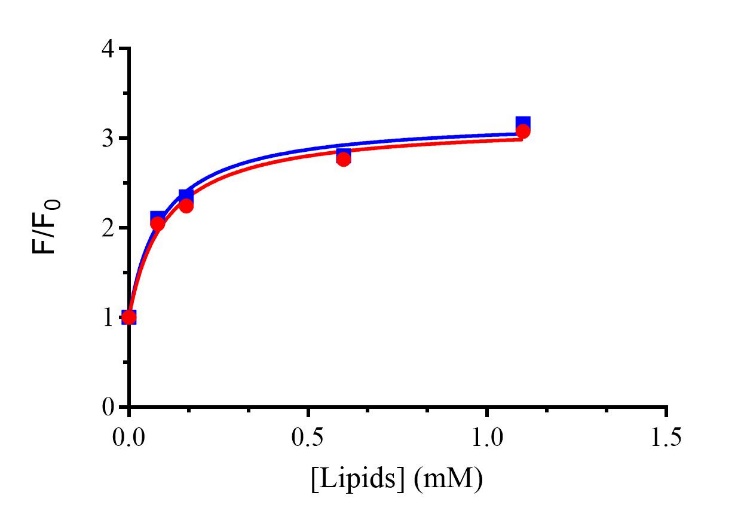
*

***Figure S1.*** *Partition isotherms for Trem-HSK (A) and Trem-HK (B) peptides in the presence of zwitterionic (red) and anionic (blue) LUVs.*


*
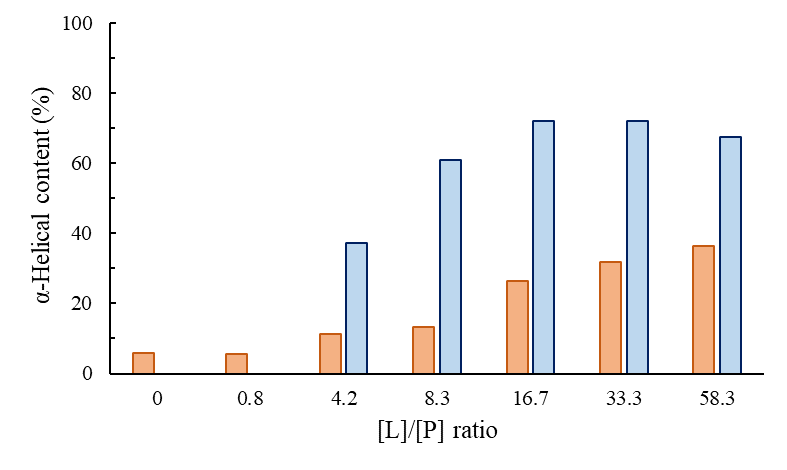
*

*
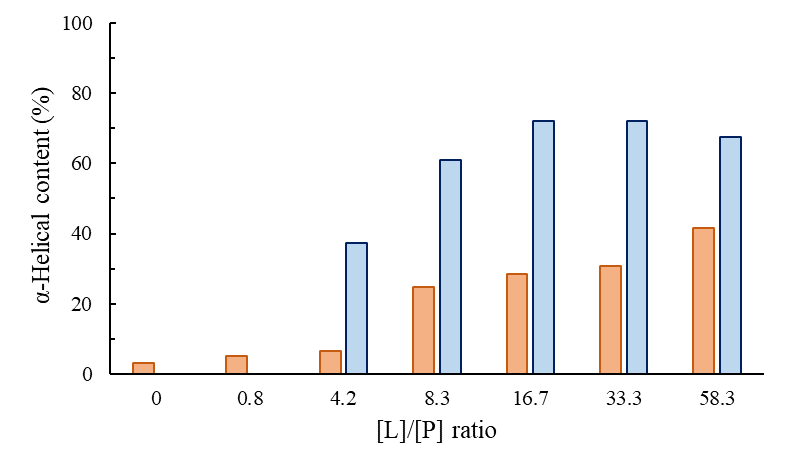
*

***Figure S2.*** *Percentage of α-helical content of Trem-HSK (A) and Trem-HK (B) peptides in the presence of POPC (orange) and (70/30)% w/w POPC - POPG (cyan) LUVs.*

*
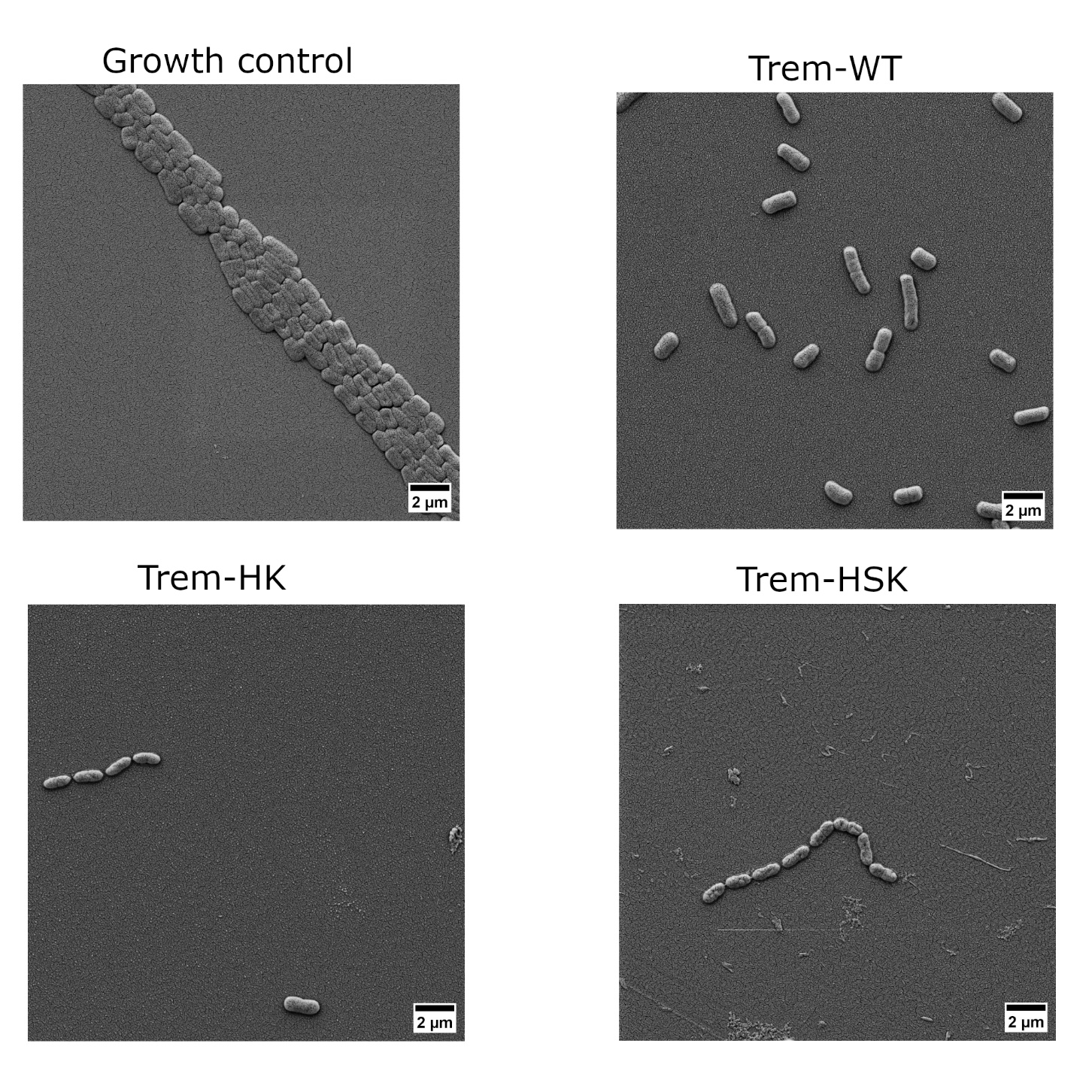
*

***Figure S3****- Scanning electron micrographs of a carbapenem-resistant K. pneumoniae cells untreated or treated with 0.75 × MIC of Trem-WT, Trem-HK and Trem-HSK for 4 hours. Magnification is 8000× and scalebar is equal to 2 µm.*

*
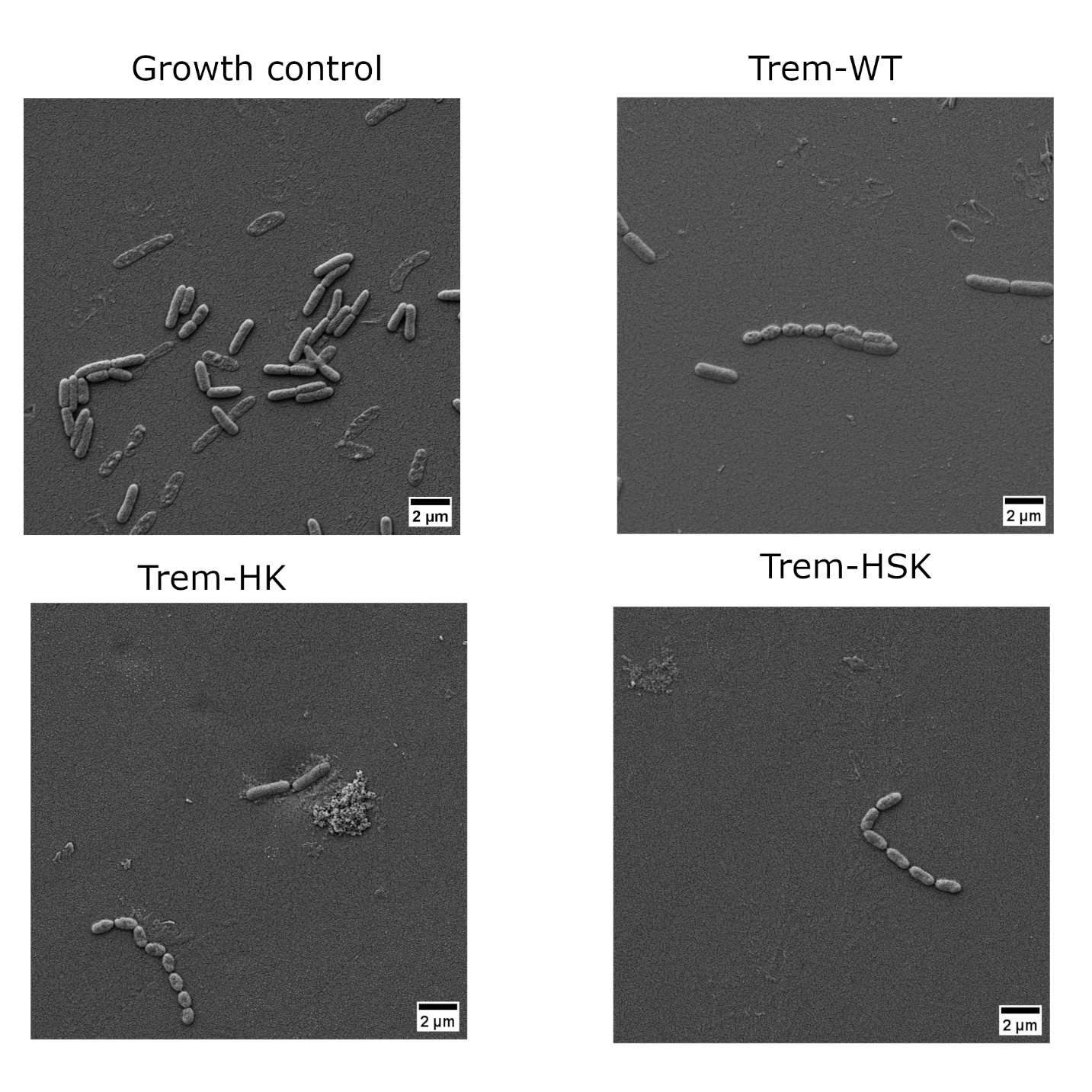
*

***Figure S4****- Scanning electron micrographs of a carbapenem-resistant P. aeruginosa cells untreated or treated with 0.75 × MIC of Trem-WT, Trem-HK and Trem-HSK for 4 hours. Magnification is 8000× and scalebar is equal to 2 µm.*

*
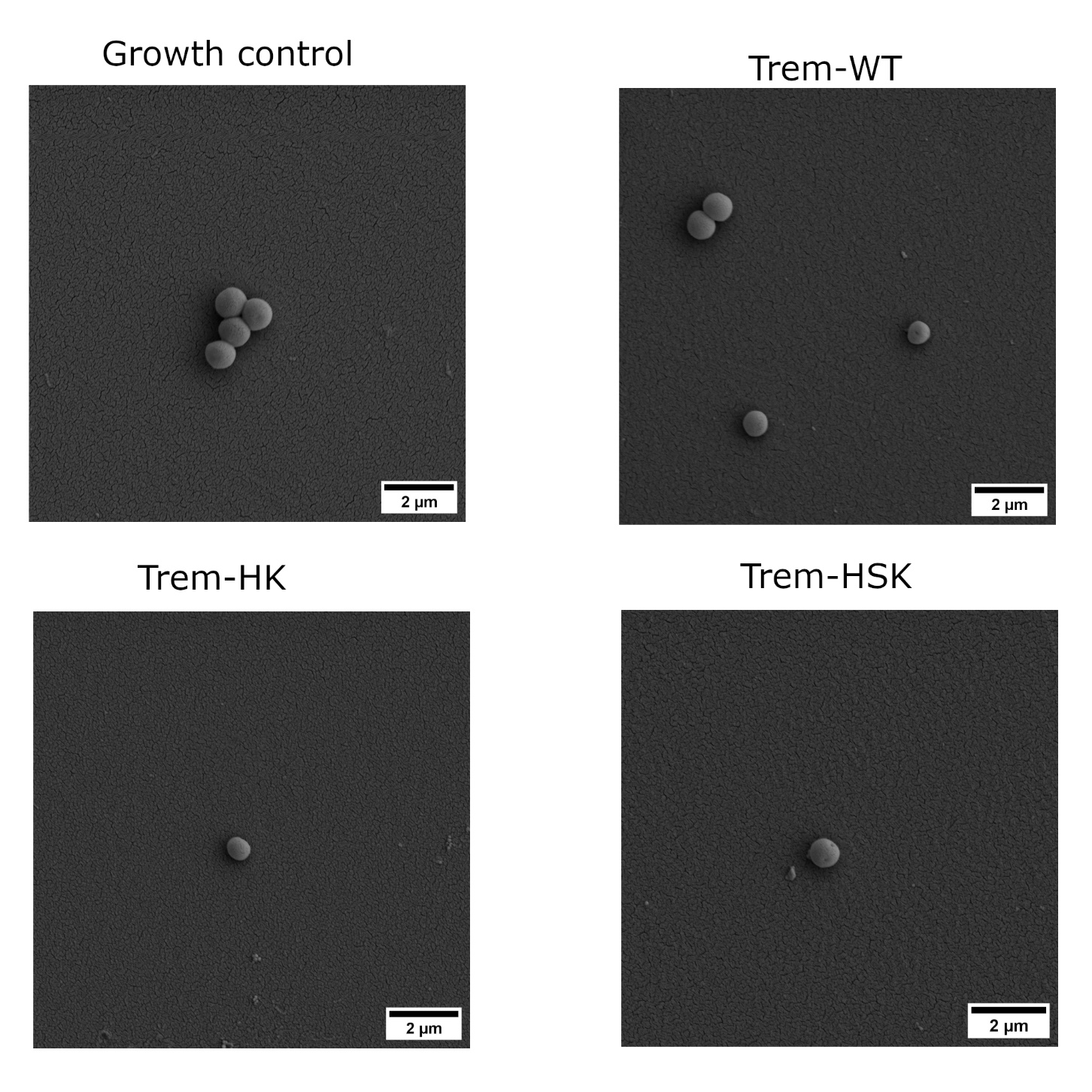
*

***Figure S5****- Scanning electron micrographs of a vancomycin-resistant E. faecium cells untreated or treated with 0.75 × MIC of Trem-WT, Trem-HK and Trem-HSK for 4 hours. Magnification is 20000× and scalebar is equal to 2 µm.*
